# Genomic analysis of clinical Aeromonas isolates reveals genetic diversity but little evidence of genetic determinants for diarrhoeal disease

**DOI:** 10.1099/mgen.0.001211

**Published:** 2024-03-07

**Authors:** Elizabeth J. Klemm, Muhammad Imran Nisar, Matt Bawn, Dilruba Nasrin, Farah Naz Qamar, Andrew Page, Farheen Qadri, Sadia Shakoor, Anita KM Zaidi, Myron M. Levine, Gordon Dougan, Robert A. Kingsley

**Affiliations:** 1 Wellcome Sanger Institute, Cambridge, UK; 2 Department of Paediatrics and Child Health, Aga Khan University, Karachi, Pakistan; 3 Quadram Institute Bioscience, Norwich, UK; 4 Faculty of Biological Sciences, University of Leeds, Leeds, UK; 5 Center for Vaccine Development and Global Health, University of Maryland School of Medicine, Baltimore, Maryland, USA; 6 Bill & Melinda Gates Foundation, Seattle, Washington, USA; 7 Cambridge Institute of Therapeutic Immunology & Infectious Disease, University of Cambridge, England, UK; 8 School of Biological Sciences, University of East Anglia, Norwich, UK

**Keywords:** *Aeromonas*, AMR, diarrhoea, genomics, GEMS, Pakistan, virulence

## Abstract

*Aeromonas* spp. are associated with a number of infectious syndromes in humans including gastroenteritis and dysentery. Our understanding of the genetic diversity, population structure, virulence determinants and antimicrobial resistance of the genus has been limited by a lack of sequenced genomes linked to metadata. We performed a comprehensive analysis of the whole genome sequences of 447 *Aeromonas* isolates from children in Karachi, Pakistan, with moderate-to-severe diarrhoea (MSD) and from matched controls without diarrhoea that were collected as part of the Global Enteric Multicenter Study (GEMS). Human-associated *Aeromonas* isolates exhibited high species diversity and extensive antimicrobial and virulence gene content. *Aeromonas caviae*, *A. dhankensis*, *A. veronii* and *A. enteropelogenes* were all significantly associated with MSD in at least one cohort group. The *maf2* and *lafT* genes that encode components of polar and lateral flagella, respectively, exhibited a weak association with isolates originating from cases of gastroenteritis.

**Impact Statement**

*Aeromonas* has been linked to diarrhoea in humans, but little is known about how it causes disease. We present the first large-scale genomic analysis of *Aeromonas* collected from children with moderate-to-severe diarrhoea (MSD) and case-matched controls. Understanding how *Aeromonas* causes disease is important as its prevalence may rise due to climate change increasing the exposure of humans to contaminated water sources where *Aeromonas* resides. We report the species diversity and variation in gene content between *Aeromonas* species, and between strains from MSD and controls. We find little evidence for genes that influence the disease phenotype, although two flagella-associated genes may play a role. *Aeromonas* isolates harboured a substantial number of antimicrobial resistance genes that have the potential to transfer to other human pathogens, which may further fuel the antimicrobial resistance crisis. This analysis provides a significant advance in our understanding of *Aeromonas* genomics that provides context for future genomic studies and hypotheses for mechanistic studies. Future studies are needed to elucidate the potential role of specific genes and host factors associated with *Aeromonas* infection.

## Data Summary

The authors confirm all supporting data, code and protocols have been provided within the article or through supplementary data files. Sequence data are available in the European Nucleotide Archive (http://www.ebi.ac.uk/ena) under project accession numbers PRJEB15489 and PRJEB1611.

## Introduction

*Aeromonas* are facultatively anaerobic Gram-negative *Gammaproteobacteria* and the genus consists of multiple species, some of which can cause disease in humans and animals [1,2]. This genus is predominantly associated with aquatic environments, including surface water, sewage and drinking water [3]. *Aeromonas* form biofilms that contribute to persistence in water systems [1] and there is growing concern that climate change may increase the exposure of humans to contaminated water sources [3]. *Aeromonas* is globally ubiquitous and has also been isolated from many other sources including fish, soil and food products [4]. Aeromonads have been shown to encode multiple putative virulence genes including secreted haemolytic toxins [5] and can harbour genes conferring antibiotic resistance, making these bacteria of broader interest for public health [4]. However, relatively little is known of the genetic diversity and population structure of aeromonads in human stool, the distribution of putative virulence factors and antimicrobial resistance genes, and how these may contribute to disease.

Humans typically acquire *Aeromonas* through ingestion of water or food, or exposure of a wound to contaminated water [4]. *Aeromonas* have been associated with necrotizing fasciitis, septicaemia, gastroenteritis and dysentery in humans [2]. The causal relationship between *Aeromonas* and diarrhoeal illness is unclear, in part because of frequent asymptomatic carriage [6]. Moreover, human challenge data showed a low incidence of diarrhoea after oral administration of large inocula of viable *Aeromonas hydrophila* to healthy adult North American volunteers [7]. The prevalence of *Aeromonas* in the human gastrointestinal tract varies based on age, geographical location, hygiene, socioeconomic conditions and nutritional status (for children) [1]. Colonization by *Aeromonas* is more common in children under 5 years of age and in areas with a tropical climate that lack improved water and sanitation facilities. A coordinated case-control study, known as the Global Enteric Multicenter Study (GEMS), of children under 5 years of age with moderate-to-severe diarrhoea (MSD) and matched controls without diarrhoea was undertaken across seven sites over a 4 year period [8]. Whereas *Aeromonas* was identified in <1 % of the children in four African sites, it was markedly more prevalent in the Karachi (Pakistan) and Mirzapur (Bangladesh) GEMS sites, identified as a significant pathogen in 22.2 % of MSD cases [9]. *Aeromonas* has also been identified as the second most commonly isolated candidate enteric bacterial pathogen in children below 18 months with gastroenteritis in Australia [10].

Advances in molecular biology and sequencing technologies have led to an improved understanding of the population structure of *Aeromonas* [2]. *Aeromonas* was originally assigned to the family *Vibrionaceae*, but 16S rRNA gene sequencing later established *Aeromonadaceae* as a distinct family [11]. Multi-locus sequence typing (MLST) of concatenated sequences of sets of housekeeping genes enabled more accurate typing and improved differentiation between closely related species. At least three MLST schemes have been proposed for *Aeromonas* [12,15]. Average nucleotide identity (ANI) provides even more accurate species delineation based on pairwise whole genome sequence comparisons, much like an *in silico* substitute for DNA–DNA hybridizations [16,17]. Such analyses have shown that many isolates of *Aeromonas dhakensis* were historically misidentified as *A. hydrophila* based on low-resolution typing techniques. These and other sequence-based approaches have shown that *Aeromonas* is an extremely diverse genus comprising many distinct species. To date, over 30 *Aeromonas* species have been identified with some species showing evidence of host and environmental preferences. Isolates from humans predominantly belong to just four species: *A. hydrophila*, *A. dhakensis*, *Aeromonas caviae* and *Aeromonas veronii* [1]. The first complete genome sequence of an *Aeromonas* isolate was published in 2006 of the type strain *A. hydrophila* ATCC 7966^T^ [18]. Since then, multiple *Aeromonas* strains have been sequenced and comparative genomics has been used to identify gene differences with attempts made to link these to phenotypes [19,21].

In this study, we present the genomic analysis of *Aeromonas* samples from children enrolled in the GEMS study in Karachi, Pakistan. These samples were taken from children under 5 years of age with MSD along with their matched controls without diarrhoea [8]. Our analysis of this large sample set linked to detailed metadata enabled us to perform a comprehensive high-resolution characterization of human-associated *Aeromonas* spp.

## Methods

### Bacterial isolates, genome sequencing, assembly and annotation

*Aeromonas* isolates were obtained from stool samples as previously described [8]. A full list of sample names and dates of isolation can be found in Table S1. Libraries for genome sequencing were prepared from genomic DNA and sequenced to generate 150 bp paired-end reads using the Illumina HiSeq System. Sequence data were submitted to the European Nucleotide Archive (http://www.ebi.ac.uk/ena); accession numbers are indicated in Table S1. The genome data were *de novo* assembled using the pipeline described [22] and annotated with Prokka (v1.5) [23], using default parameters. Reference sequences were downloaded from the PATRIC database (www.patricbrc.org/). Strains JTBH, JTBG and JTBK were re-assembled to correct for pseudogenes. Pacbio read-data were downloaded and assembled using the Pacbio SMRT analysis pipeline (v2.3) (https://smrt-analysis.readthedocs.io/en/latest/SMRT-Pipe-Reference-Guide-v2.2.0/. Following first-stage unfinished assembly, JTBH had two polished contigs totalling 4 572 25 bp, JTBG had six contigs totalling 4 906 303 bp and JTBK had one contig with a length of 4 534 321 bp. The assemblies were then checked using Miniasm [24], re-orientated to begin at the *thrA* gene, circularized using Minimus [25] and annotated using Prokka [23], using default parameters.

### Comparison of average nucleotide identity

ANI was calculated using the ANIm (MUMmer) method with pyANI, https://github.com/widdowquinn/pyani. Scores above 96 % were classed as indicating the same species. As has been previously reported, we found some discrepancies in the species designations in the databases (such as *A. dhakensis* samples labelled as *A. hydrophila*). For these discrepencies, we used the predominant species labelling within each group. We additionally found a sample labelled *A. veronii* AMC34 that is classified as *A. veronii* but meets the cutoff for classification as a distinct species.

### Pan-genome and phylogenetic analyses

A pan-genome was created using Roary (v3.7.0) [26] with an identity of 80 %, which resulted in 27 119 gene clusters and otherwise default settings. There were 2520 core genes covering 1 868 773 bases out of approximately 4.5 Mb for *Aeromonas* on average. The core gene alignment consisted of 714 947 SNP sites, calculated using SNP-sites (v2.3.2) [27]. The core gene multi-FASTA alignment was used to reconstruct a phylogenetic tree with fasttree using the GTRGAMMA model, 100 bootstraps and otherwise default settings. Phylogenetic trees were visualized with iTOL (v3) [28]. Principal component analysis of presence and absence of genes in the accessory genome was performed with the prcomp function in R.

### Gene identification from short-read sequence data

To identify virulence genes, a manually curated list of known virulence genes from *Aeromonas* was constructed consisting of entries in the Virulence Factors Database [29] supplemented with other genes identified from the literature (File S1). A single representative nucleotide sequence from each cluster in the pan-genome was compared to the manually curated list of virulence genes using blastn (v2.4.0) [30]. The results were filtered to include only hits where the query coverage was >70 % and the identity of the match was >90 %, which removed low-quality partial matches. This gave the presence and absence of each virulence gene in each isolate. To identify antibiotic resistance genes in Illumina short-read sequence data, ARIBA software was run on the FASTQ files with the CARD database [31] using a maximum divergence cut-off of 30 % and coverage >90 %. Data were visualized using phandango [32].

### Statistical analysis of association of *Aeromonas* species candidate virulence genes and MSD

The workflow and exclusion of samples is summarized in Fig. S5. Of 3096 enrolments from Pakistan (1258 cases and 1838 controls), *Aeromonas* was detected in 493 samples. Genomic data were available for 447 (244 cases and 203 controls). We performed logistic regression analysis to test for an association of 226 candidate virulence genes with MSD. We excluded genes that were universally present or had a frequency of less than five from the analysis. For 136 genes we performed a chi square test of association. Multivariate analysis was then performed for 34 genes with a *P*-value of ≤0.2. Four models were constructed using a backward elimination process in which genes that had a *P*-value >0.05 were removed in a stepwise fashion in order to arrive at the best fitting model with the fewest variables. A multivariate model adjusted for the presence of all other genes, a model additionally adjusted for presence of all other pathogens detected in the original study, a model adjusted for sociodemographic characteristics, and finally a model adjusted for all other genes, sociodemographic factors and presence of other pathogens. A *P*-value of <0.05 was considered significant.

## Results

### Whole genome sequencing of *Aeromonas* samples isolated from children in Karachi, Pakistan, reveals a high level of species diversity with an open accessory genome

In GEMS, stool samples were taken from children aged 0–59 months with MSD and from age- and gender-matched controls without diarrhoea [8]. The whole genome sequences of 447 *Aeromonas* isolated in Karachi, Pakistan during GEMS, were determined (accession numbers in Table S1, available in the online version of this article), revealing an average genome size of approximately 4.5 Mb. To determine the species of each isolate, we calculated the ANI score and compared each isolate from this study and *Aeromonas* whole genome sequences in available databases. We used a stringent cut-off of 96 % to delineate species (Table S2) and found 287 *A*. *caviae*, 86 *A*. *veronii*, 44 *A*. *dhakensis*, 22 *A*. *enteropelogenes* (also called *A. trota*), and eight from other *Aeromonas* species including one isolate representing a novel species with <96 % ANI compared to genomes of previously described species (Table S3). Diversity was greatest within *A. veronii* with an average ANI value of 96.43 %. The species observed generally concur with those previously suggested as being associated with human disease, with the notable exception of the general absence of *A. hydrophila*.

**Fig. 1.**
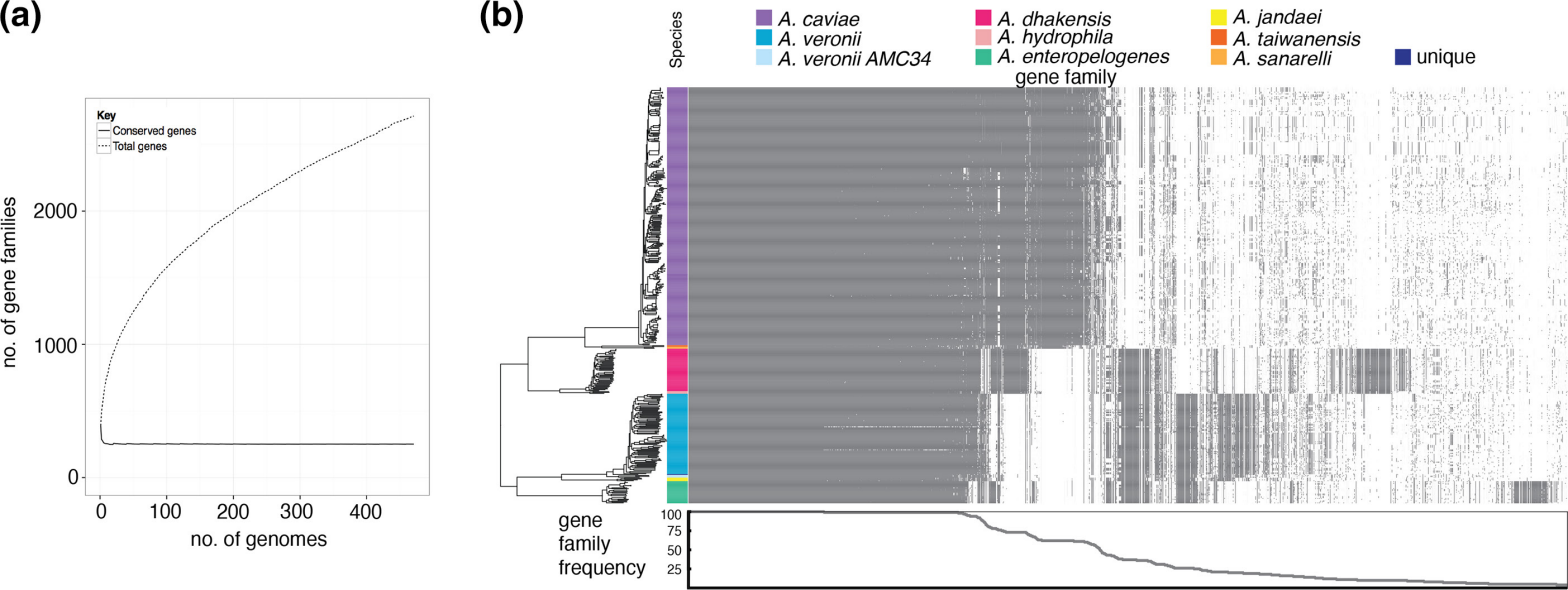
Pan-genome of *Aeromonas* species isolated from children in Pakistan. (**a**) Cumulative number of genes plotted against the number of *Aeromonas* genomes. (**b**) Gene content of *Aeromonas* isolates (rows), and presence of individual genes (columns) indicated by grey coloration. Plot at the bottom represents percentage of isolates encoding a specific gene.

The pangenome of this set comprised 27 119 gene families at ≥80 % amino acid sequence identity. Of these, 2520 are identified as core genes present in all isolates, constituting a concatenated coding sequence length of 1 868 773 bp. The pangenome is ‘open’ in that additional genomes are predicted to increase the size of the pangenome (Fig. 1a). Groups of genes in the accessory genome clustered with the different species (Fig. 1b).

**Fig. 2.**
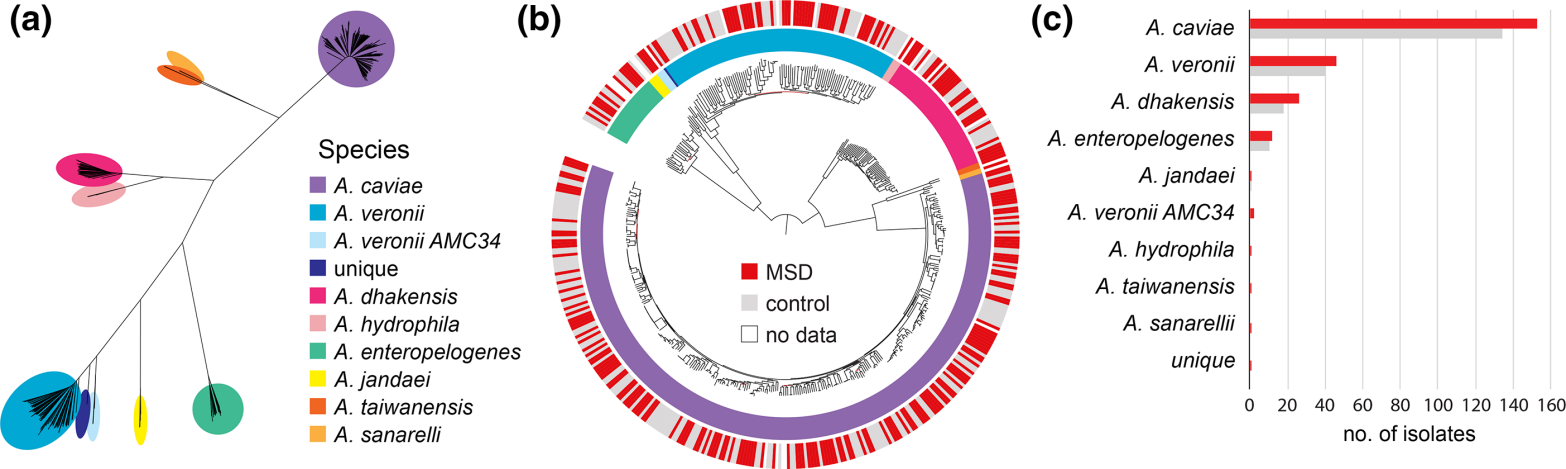
Population structure of *Aeromonas* isolates from human stool samples in Pakistan. (**a**) An unrooted maximum-likelihood phylogenetic tree of 447 Pakistan isolates and 24 reference genomes inferred from 714 947 SNPs in the core genome. (**b**) Midpoint-rooted tree with species (inner ring) colours from (**a**) and disease status (outer ring) with red branches indicating internal nodes with <70 % bootstrap confidence. (**c**) Number of *Aeromonas* isolates from diarrhoea cases and controls for each species.

A maximum-likelihood phylogenetic tree was reconstructed based on the 714 947 SNPs present in the core genome. Long branches in the tree distinguished the isolates according to species inferred from ANI (Fig. 2a). Moreover, the topology of the tree was highly supported with over 90 % of the nodes having high (>70 %) bootstrap confidence. Most of the lower bootstrap confidence nodes were in the shorter branch nodes. The majority of the terminal branch lengths were long, indicating that even the nearest neighbours were genetically diverse. This indicated that these *Aeromonas* samples did not represent outbreaks of infection, but were more probably acquired by the children from a diverse *Aeromonas* population in the sample region environment. Only a few lineages had short terminal branches, indicating closer genetic similarity (Fig. 2b). Most of the closely related isolates were of *A. caviae*.

### Little evidence of disease causality associated with specific species

Overall, 244 of the 447 *Aeromonas* isolates were from cases of MSD, equating to ~55 %, and the rest were isolated from matched controls without diarrhoea. Within each significantly represented species (>20 isolates), we found approximately the same percentage (53–59 %) derived from MSD cases as from isolates from control group children, suggesting that there is not an association between particular species and MSD (Table S3 and Fig. 2c). Isolates associated with MSD were distributed across the tree and did not appear in greater frequency among any of the different lineages (Fig. 2b). Moreover, even in a limited number of cases where isolates were very closely related and isolated over a similar timeframe (June to August 2009), there was commonly a lack of concordance in diarrhoeal status of the child (Fig. S1). We performed principal component analysis (PCA) on isolates from the most common species to determine whether the composition of the accessory genome correlated with disease status, but found no such association (Fig. S2).

Previously, an association of MSD with the presence of *Aeromonas* was investigated using conditional logistic regression analyses at the genus level, since species of isolates was not determined [8]. We therefore extended this analysis to investigate the association of *A. caviae*, *A. dhankensis*, *A. veronii* and *Aeromonas enteropelogenes* species with MSD in three age strata in Pakistan (Table 1). *A. caviae*, *A. dhakensis* and *A. veronii* were each associated (*P*<0.05) with MSD in at least one age stratum, while *A. enteropelogenes* was not in any age strata. For *A. caviae*, MSD cases had two times the odds [odds ratio (OR): 2.0, confidence interval (CI): 1.31–3.03] in the 0–11 months age range and three times the odds (3.0, CI: 1.85–4.96) in the 24–59 month age range of carrying this species than control cases. For *A. dhakensis*, in the 24–59 months age range MSD cases had three times the odds (OR: 3.0, CI: 1.85–4.96) of carrying *A. caviae* than control cases. For *A. veronii*, in the 24–59 months age range MSD cases had over three times the odds (OR: 3.2, CI: 0.94–3.63) of carrying *A. caviae* than control cases. In the 12–23 months age range, no specific species was significantly associated (*P*<0.05) with MSD over control isolates.

**Table 1.** Association of *Aeromonas* species with moderate and severe diarrhoea in Pakistan

		**Controls**	**Cases**	**Total**		
**Age strata0–11 months**		***N*=630**	***N*=615**	***N*=1245**	**OR (CI**)	***P*** **-value**
*Aeromonas* spp.	No	574 (91.1 %)	516 (83.9 %)	1090 (87.5 %)	2 (1.39, 2.86)	0.000
	Yes	56 (8.9 %)	99 (16.1 %)	155 (12.4 %)		
*A. caviae*	No	591 (93.8 %)	545 (88.6 %)	1136 (91.2 %)	2 (1.31, 3.03)	0.001
	Yes	39 (6.2 %)	70 (11.4 %)	109 (8.8 %)		
*A.dhakensis*	No	621 (98.6 %)	606 (98.5 %)	1227 (98.5 %)	1 (0.39, 2.52)	1.000
	Yes	9 (1.4 %)	9 (1.5 %)	18 (1.5 %)		
*A.veronii*	No	625 (99.2 %)	599 (97.4 %)	1224 (98.3 %)	3.2 (1.17, 8.73)	0.023
	Yes	5 (0.8 %)	16 (2.6 %)	21 (1.7 %)		
*A.enteropelogenes*	No	628 (99.7 %)	612 (99.5 %)	1240 (99.6 %)	1.5 (0.25, 8.97)	0.657
	Yes	2 (0.3 %)	3 (0.5 %)	5 (0.4 %)		
**Age strata 12–23** **months**		***N*=672**	***N*=394**	***N*=1066**		
*Aeromonas* spp.	No	589 (87.6 %)	316 (80.2 %)	905 (84.9 %)	1.9 (1.34, 2.73)	0.000
	Yes	83 (12.4 %)	78 (19.8 %)	161 (15.1 %)		
*A.caviae*	No	619 (92.1 %)	353 (89.6 %)	972 (91.2 %)	1.4 (0.93, 2.25)	0.095
	Yes	53 (7.9 %)	41 (10.4 %)	94 (8.8 %)		
*A.dhakensis*	No	666 (99.1 %)	384 (97.5 %)	1050 (98.5 %)	2.7 (0.97, 7.57)	0.055
	Yes	6 (0.9 %)	10 (2.5 %)	16 (1.5 %)		
*A.veronii*	No	654 (97.3 %)	376 (95.4 %)	1030 (96.7 %)	1.8 (0.94, 3.63)	0.070
	Yes	18 (2.7 %)	18 (4.6 %)	36 (3.4 %)		
*A.enteropelogenes*	No	666 (99.1 %)	389 (98.7 %)	1055 (99 %)	1.4 (0.39, 5.08)	0.585
	Yes	6 (0.9 %)	5 (1.3 %)	11 (1 %)		
**Age strata 24–59** **months**		***N*=524**	***N*=215**	***N*=739**		
*Aeromonas* spp.	No	460 (87.8 %)	148 (68.8 %)	608 (82.3 %)	3.2 (2.15, 4.88)	0.000
	Yes	64 (12.2 %)	76 (31.2 %)	131 (17.7 %)		
*A.caviae*	No	482 (92 %)	173 (80.5 %)	655 (88.6 %)	3.0 (1.85, 4.96)	0.000
	Yes	42 (8 %)	42 (19.5 %)	84 (11.4 %)		
*A.dhakensis*	No	521 (99.4 %)	208 (96.7 %)	729 (98.7 %)	4.7 (1.19, 19.22)	0.027
	Yes	3 (0.6 %)	7 (3.3 %)	10 (1.3 %)		
*A.veronii*	No	507 (96.8 %)	203 (94.4 %)	710 (96.1 %)	1.7 (0.79, 3.70)	0.167
	Yes	17 (3.24 %)	12 (5.6 %)	29 (3.9 %)		
*A.enteropelogenes*	No	522 (99.6 %)	211 (98.1 %)	733 (99.2 %)	4.4 (0.80, 25.12)	0.087
	Yes	2 (0.4 %)	4 (1.9 %)	6 (0.8 %)		

### Limited evidence that virulence-associated gene presence was associated with moderate and severe diarrhoea

**Fig. 3.**
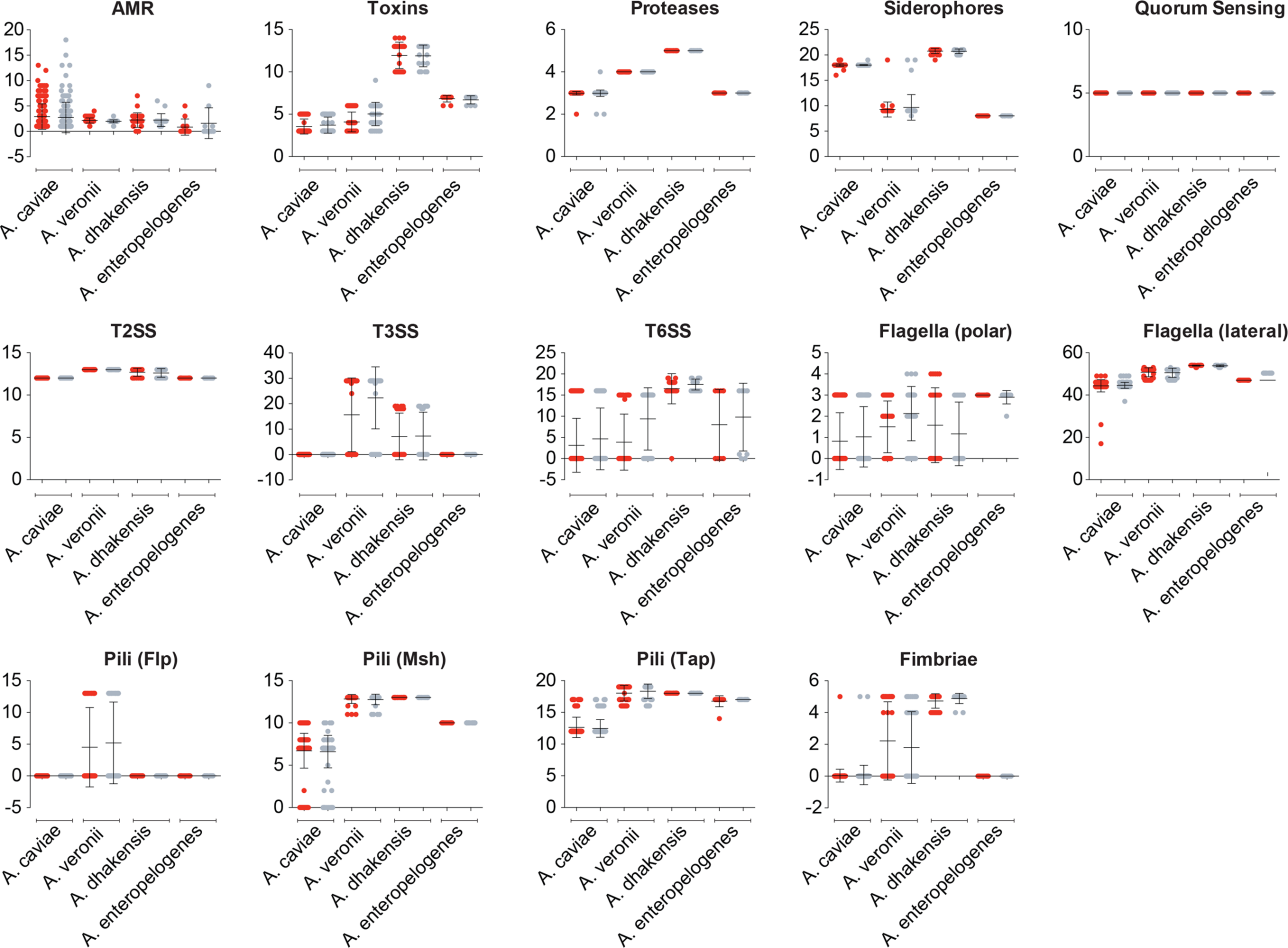
Inter- and intra-species variation in gene content. Number of genes belonging to antimicrobial resistance (AMR), toxin, protease, siderophore, quorum sensing, type II secretion systems, type III secretion systems, type VI secretion systems, polar flagella, lateral flagella, pili and fimbria functional classes for isolates from diarrhoea cases (red circles) and control cases (grey circles). The mean (horizontal lines) and standard deviation (vertical lines) are indicated.

Although overall accessory genome composition was not associated with disease causality in these samples, we explored the possibility that individual genes could be responsible for differences in clinical outcome. We tested the hypothesis that the isolates from children with MSD were enriched with known virulence-associated factors. We compiled a list of 136 genes that have been previously implicated with virulence in *Aeromonas* and related species, including those encoding toxins, secretion systems (type 2, T2SS; type 3, T3SS; type 6, T6SS), effector proteins, proteases, siderophore transport, quorum sensing, lateral and polar flagella biosynthesis, pili and fimbriae. A database of a representative sequence of each of the genes was constructed (File S1) and used to query their presence in the isolates (Table S4). The results were plotted and mapped to the phylogeny (Fig. S3). There was generally no significant difference in the numbers of genes from the different functional classes observed between diarrhoea and control isolates from the same species (Fig. 3). The only exception was for *A. veronii*, for which the control isolates had significantly greater numbers of T3SS, T6SS and toxin genes compared to the diarrhoea isolates (*P*<0.005, Tukey test). We did, however, observe differences in gene content based on species, with *A. dhakensis* harbouring more toxin, T6SS and fimbriae genes compared to the other species. Indeed, by mapping the virulence-associated gene presence to the phylogenetic tree, it was clear that several gene sets were inherited along specific branches, for example T3SS genes in *A. dhakensis* and *A. veronii*, Flp pili genes in *A. veronii* (Fig. S3B) and lateral flagellum genes in *A. caviae*, *A. dhakensis* and *A. veronii* (Fig. S3C).

We next carried out a logistic regression analysis to test for an association with MSD of 136 candidate virulence genes that were not universally present or had a frequency of five or more, using a chi square test of association (Table S5). Genes with *P*≤0.2 were further subjected to multivariate analysis. Three models were used to adjust for the presence of all other candidate virulence genes, presence of all other pathogens detected in the GEMS study and sociodemographic characteristics, and finally a model adjusted for all these factors (Table S6). Two genes, *lafT* and *maf2*, encoding products affecting lateral or polar flagella biogenesis [33,34] were significantly associated (*P*<0.05) with MSD in all models considered. The *lafT* gene was present within multiple subclades of all four major species with an overall representation in *A. caviae* (31 %), *A. dhakensis* (43 %), *A. enteropelogenes* (100 %) and *A. veronii* (70 %). In contrast, the *maf2* gene was limited to *A. dhakensis* (100 %) and *A. veronii* (5 %) (Fig. S3C). Four genes, *ascB* encoding a putative chaperone protein of the T3SS [35], AHA1843 and AHA2456 encoding putative components of a T6SS, and *vgrG* encoding a T6SS toxin, were significantly negatively associated (*P*<0.05) with MSD after adjusting using at least one of the models (Table S6). The *acsB* gene was only present in isolates of *A. dhakensis* and *A. veronii* while the T6SS genes had similar distribution with the notable absence of AHA184 in *A. veronii* isolates (Fig. S3B). The *vgrG* gene was present in all isolates of *A. dhakensis* and *A. enteropelogenes,* and in a minority of *A. caviae* (31 %) and *A. veronii* (42 %) (Fig. S3A).

### Aeromonads harbour a high level of AMR genes

Aeromonads occupy environmental niches known for antibiotic exposure and we explored the possibility that they could be a potential reservoir for antimicrobial resistance (AMR) determinants. We screened the isolates to identify their repertoire of AMR genes. In total, 96% of the isolates (429 out of 447) encoded at least one AMR gene and, on average, each isolate harboured 2.5 AMR genes (Fig. S4). There was no statistically significant difference in the number of AMR genes distributed between isolates from MSD cases compared to isolates from control group children, with respect to each species. Eight isolates had ten or more individual AMR genes, showing the potential for *Aeromonas* spp. to acquire a large number of AMR genes. Indeed, 114 out of 447 isolates (26 %) can be classified as multi-drug resistant (MDR) because they have the genotypic potential for resistance to three or more classes of antimicrobials. *A. caviae* had the greatest percentage of predicted MDR isolates with 103 out of 287, or 36 %, whilst *A. enteropelogenes* had 18 % and *A. dhakensis* had 11 %; *A. veronii* had only one predicted MDR isolate out of 86. The most common genes were β-lactamases, with *A. caviae* frequently harbouring *mox* (251 out of 287 isolates, 87 %), and *A. dhakensis* and *A. veronii* frequently harbouring *oxa-12 (*59 and 95 %, respectively) and *cphA2* (95 and 99 %, respectively)*.* The *cphA* gene has been previously reported to be intrinsically harboured by *A. veronii* and *A. dhakensis* [36]. Some AMR genes were associated with specific lineages. For example, *aac(6')-lld*, *blaCMY-1* and *sul1* are carried by most isolates within a specific lineage of *A. caviae* and could indicate the presence of the same mobile genetic element carrying these genes. Similarly, another specific lineage of *A. caviae* is associated with the carriage of *tetE* (Fig. S4).

## Discussion

Herein we describe a comprehensive genomic analysis of a large number of *Aeromonas* isolates from children with or without diarrhoea in Karachi, Pakistan, isolated as part of GEMS [8]. The large genetic diversity observed in these geographically restricted *Aeromonas* isolates and paucity of closely related isolates suggest that they represent a subset of aeromonads present in the local environment. While only a few *Aeromonas* species are associated with humans, multiple lineages within the different species can colonize the human gut. Contrary to previous reports, we observed very few *A. hydrophila* and many *A. dhakensis* isolates in these human-derived samples [37]. This discrepancy may be due to the use of whole genomes as part of the species assignment methodology, which has higher accuracy and resolution than other typing techniques. Consistent with a report of *Aeromonas*-associated gastroenteritis in Brazil [38], the largest proportion of isolates were of *A. caviae* and *A. veronii*.

*Aeromonas* spp. were previously found to be associated with MSD in Pakistan and Bangladesh, particularly in the presence of *Shigella* and stunting of the patient [8]. Nonetheless, the association with MSD remained following adjustment for the presence of other pathogens and socioeconomic factors [9]. We found that the odds of the main four *Aeromonas* species, except *A. enteropelogenes*, had a statistically significant increase in at least one of the age strata when MSD was present. The relatively low frequency of detection of some species may have limited our ability to detect some associations. We also observed some possible evidence of differential association of species within different age strata. For example, the OR for *A. caviae*, *A. dhakensis* and *A. enteropelogenes* was greatest in the oldest cohort (24–59 months) and the OR for *A. veronii* was greatest in the youngest cohort (0–11 months). The significance of these differences requires further investigation, but may reflect a distinct ability of *Aeromonas* species to exploit the metabolic niche in the intestine of each age stratum cohort due to differences in gut microbiota or nutrient availability [39].

Many bacteria considered pathogenic are associated with specific lineages or have specific genetic elements that are believed to be responsible for the virulent phenotype. For example, pathogens may fall into defined phylogenetically distinct lineages or encode clusters of virulence-associated factors such as adhesins, effectors or toxins [40]. Two genes, *maf2* and *lafT,* were associated with MSD after adjusting for the presence of all other candidate virulence genes or socioeconomic factors in a multivariate analysis. The *maf2* and *lafT* genes encode non-structural components associated with polar or lateral flagella function or biogenesesis, and LafT has been implicated in lateral flagella-mediated adhesion of *A. hydrophila* to enterocytes [33,34]. The function of the Maf-2 protein is not known but mutants failed to develop both polar and lateral flagella, although unglycylated flagellin subunits could still be detected [33]. LafT has been proposed to function as a flagella motor protein [34], implicating lateral flagella-mediated motility in infection. The *maf2* gene was restricted to *A. dhakensis* and just six isolates of *A. veronii*, and *lafT* was only sporadically present within the genus *Aeromonas*, and many *Aeromonas* strains isolated from cases of MSD did not encode either of these genes. These data provide the rationale for further investigation of the role of these genes in the pathogenesis of *Aeromonas* in suitable models of infection. In contrast to *maf2* and *lafT*, two genes encoding components of a T6SS and a gene encoding a component of a type III secretion system [35] were moderately but statistically significantly negatively associated with MSD. This is perhaps surprising since similar macromolecular structures play important roles in the pathogenesis in other enteric bacteria, and the T6SS spike-like trimer protein VgrG of *A. hydrophila* has been reported to have toxin activity [41]. One possibility to account for this difference is that these virulence genes could be associated with other disease presentations associated with *Aeromonas* outside of the human gastrointestinal tract.

Other factors beyond the genotype may also contribute to diarrhoeal disease associated with *Aeromonas*. For example, we cannot rule out differences in expression of *Aeromonas* virulence factors or other genes under epigenetic control and therefore not detectable by DNA sequencing. The status of the host including genetic background, malnutrition level, immunity and microbiota composition may also determine the disease outcome. Co-infections with other bacteria or viruses may also account for why some *Aeromonas* isolates are associated with MSD and others are not. For example, blooms of *Proteobacteria* have been observed in the inflamed gut [42]. In this way, *Aeromonas* may be an opportunistic pathogen or incidental to dysbiosis, infection or inflammation. Thus, more studies are needed if we are to understand the association of *Aeromonas* with diarrhoea in children.

Genomic sequencing of *Aeromonas* from these samples revealed an ability to carry large number of antibiotic resistance genes, with a small number of isolates in this study carrying over ten AMR genes. The presence of *Aeromonas* in environmental niches shared by other enteric pathogens is troubling as they may serve as a reservoir for antibiotic resistance genes that could be transferred to pathogenic bacteria. More work is thus required to assess the content of AMR genes in *Aeromonas* samples from the environment and the possibility for their transfer to other bacterial species, especially if they are carried on plasmids or mobile elements. Plasmid types and the genomic context of the AMR genes were not investigated in this study.

In summary, this genome analysis of an important sample set from a defined setting provides the most comprehensive insight into the population structure of *Aeromonas* bacteria in children to date. These data should help direct further investigations either in the field or in the laboratory. For example, investigation of the relationship between the gut microbiota, *Aeromonas* colonization and diarrhoea may shed further light on the aetiology of this disease.

## supplementary material

10.1099/mgen.0.001211 Uncited Supplementary Material 1.

10.1099/mgen.0.001211 Uncited Table S1.

## Abbreviations

AMR: antimicrobial resistance
ANI: average nucleotide identity
CI: confidence interval
GEMS: Global Enteric Multicenter Study
MLST: multi-locus sequence typing
MSD: moderate-to-severe diarrhoea
OR: odds ratio
PCA: principal components analysis
rRNA: Ribosomal RNA
T2SS: type II secretion system
