# Supplementary material for: Genomic analysis of clinical Aeromonas isolates reveals genetic diversity but little evidence of genetic determinants for diarrhoeal disease
Source: Microb Genom. 2024 Mar 7;10(3):001211. doi: 10.1099/mgen.0.001211 (PMC10999740; doi:10.1099/mgen.0.001211)
Supplement: Uncited Supplementary Material 1. [file mgen-10-01211-s001.pdf]

Supplementary Material

Genomic analysis of clinical *Aeromonas* isolates reveals genetic diversity but little evidence of genetic determinants for diarrhoeal disease

Elizabeth J Klemm[1,#], Muhammad Imran Nisar[2,#], Matt Bawn[3], John Lees[1], Dilruba Nasrin[4], Farah Naz Qamar[2], Andrew Page[3], Farheen Qadri[z], Sadia Shakoor[2], Anita KM Zaidi[2,5], Myron M Levine[4,*], Gordon Dougan[6,*] and Robert A Kingsley[3,7,*]

[1] Wellcome Sanger Institute, Cambridge, UK

[2] Department of Paediatrics and Child Health, Aga Khan University, Karachi, Pakistan

[3] Quadram Institute Bioscience, Norwich, UK

[4] Center for Vaccine Development and Global Health, University of Maryland School of Medicine, USA

[5] Bill & Melinda Gates Foundation, Seattle, USA

[6] Cambridge Institute of Therapeutic Immunology & Infectious Disease, University of Cambridge, UK

[7] School of Biological Sciences, University of East Anglia, UK

# These authors contributed equally

* These authors contributed equally

Figure S1

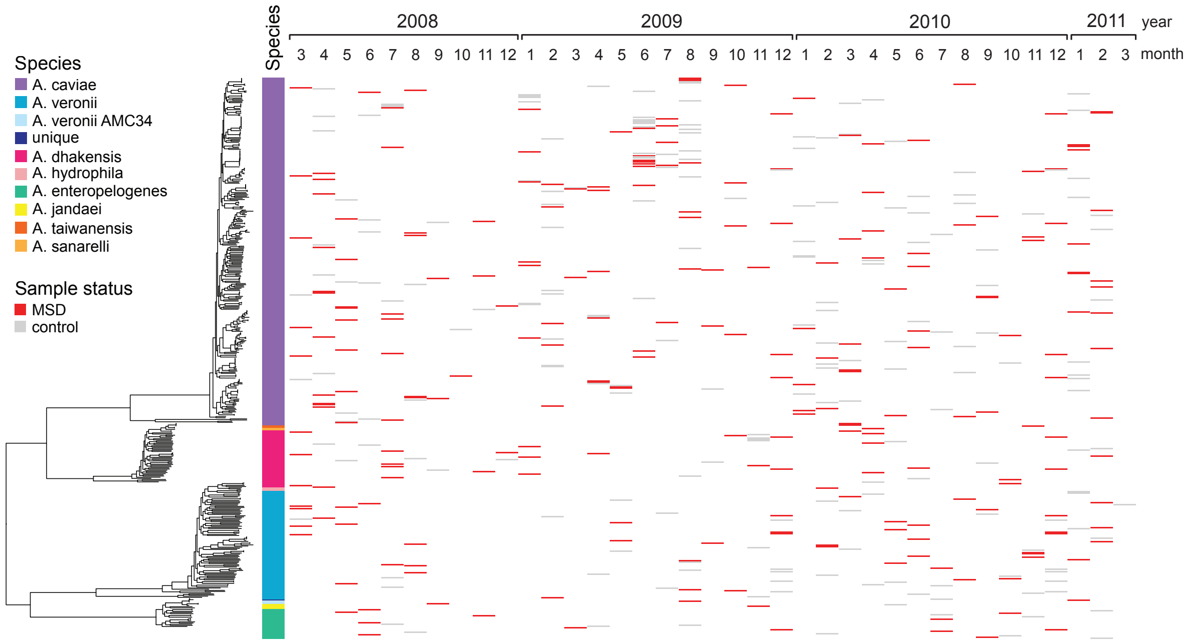

Timeline of Aeromonas isolates from Karachi, Pakistan Phylogeny from Figure 2 with month of isolation indicated. Isolates from diarrhoea cases in red and control cases in gray.

Figure S2. PCA plots based on the presence of accessory genes Isolates from diarrhoea cases in red, control cases in gray, and references in black.

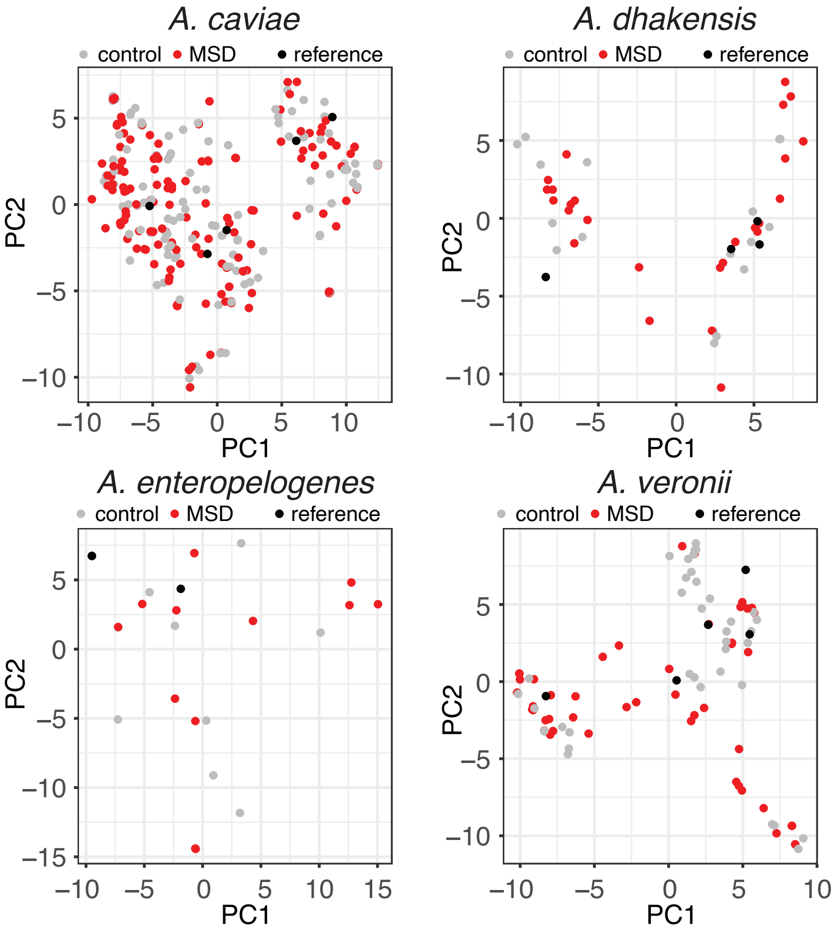

Figure S3 **Presence of virulence genes mapped on phylogenetic trees**

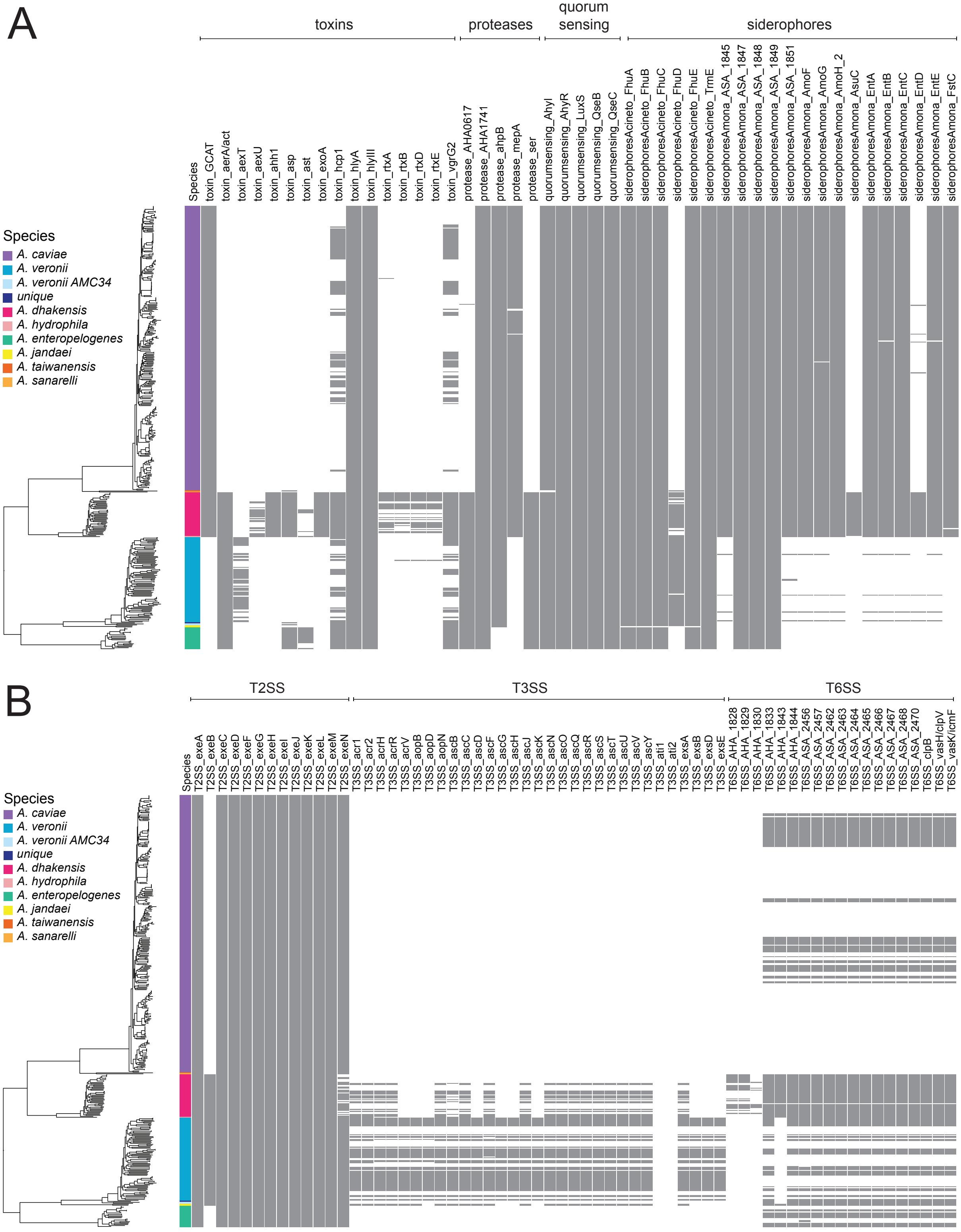

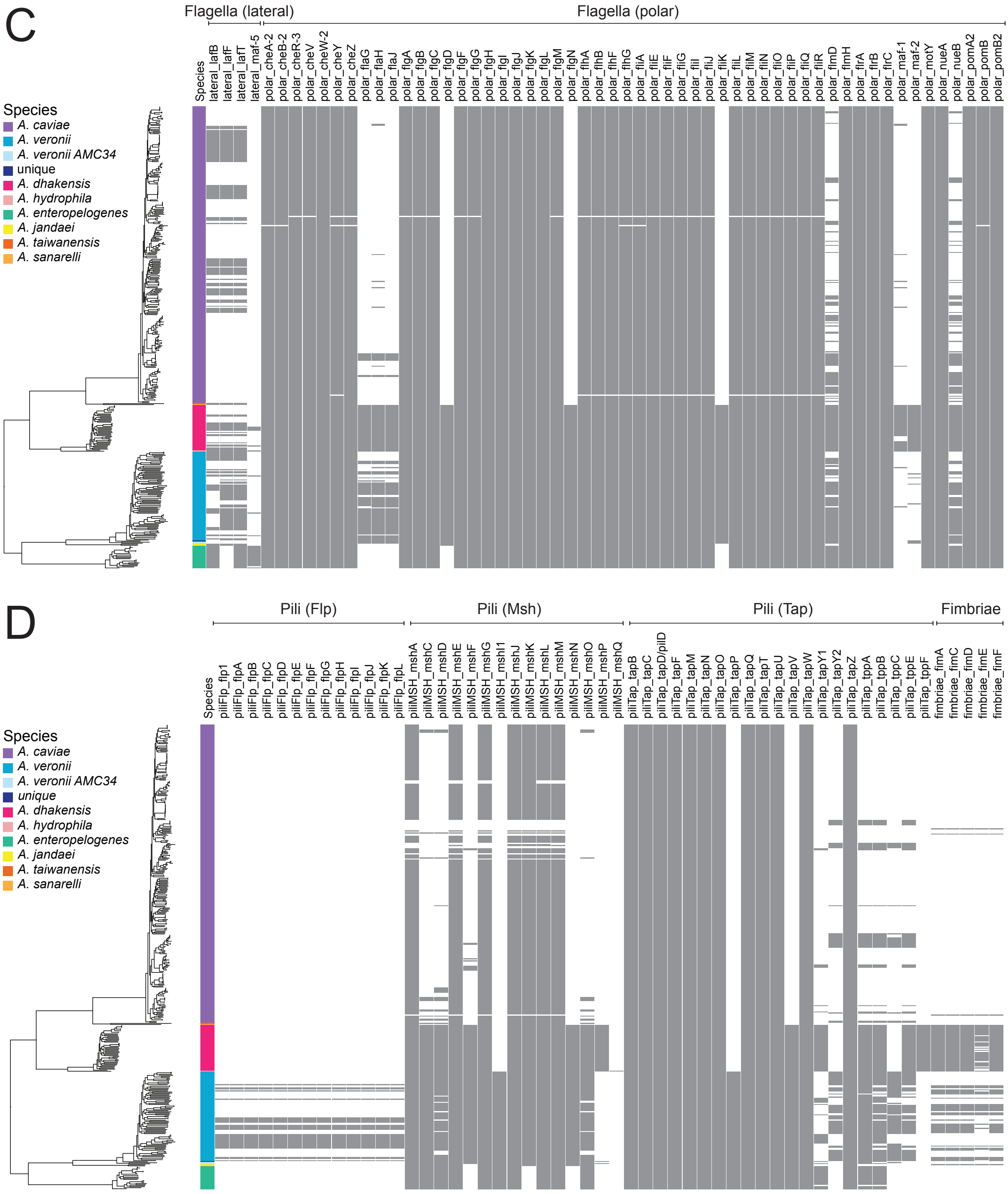
C
Flagella (lateral)
Flagella (polar)
Species
lateral_lafB
lateral_lafF
lateral_lafT
lateral_maf-5
polar_cheA-2
polar_cheB-2
polar_cheR-3
polar_cheV
polar_cheW-2
polar_cheY
polar_cheZ
polar_flaG
polar_flaH
polar_flaJ
polar_flgA
polar_flgB
polar_flgC
polar_flgD
polar_flgF
polar_flgG
polar_flgH
polar_flgI
polar_flgJ
polar_flgK
polar_flgL
polar_flgM
polar_flgN
polar_flhA
polar_flhB
polar_flhF
polar_flhG
polar_fliA
polar_fliE
polar_fliF
polar_fliG
polar_fliI
polar_fliJ
polar_fliK
polar_fliL
polar_fliM
polar_fliN
polar_fliO
polar_fliP
polar_fliQ
polar_fliR
polar_flmD
polar_flmH
polar_flrA
polar_flrB
polar_flrC
polar_maf-1
polar_maf-2
polar_motY
polar_nueA
polar_nueB
polar_pomA2
polar_pomB
polar_pomB2
Species
A. caviae
A. veronii
A. veronii AMC34
unique
A. dhakensis
A. hydrophila
A. enteropelogenes
A. jandaei
A. taiwanensis
A. sanarelli
D
Pili (Flp)
Pili (Msh)
Pili (Tap)
Fimbriae
Species
piliFlp_flp1
piliFlp_flpA
piliFlp_flpB
piliFlp_flpC
piliFlp_flpD
piliFlp_flpE
piliFlp_flpF
piliFlp_flpG
piliFlp_flpH
piliFlp_flpI
piliFlp_flpJ
piliFlp_flpK
piliFlp_flpL
piliMSH_mshA
piliMSH_mshC
piliMSH_mshD
piliMSH_mshE
piliMSH_mshF
piliMSH_mshG
piliMSH_mshI1
piliMSH_mshJ
piliMSH_mshK
piliMSH_mshL
piliMSH_mshM
piliMSH_mshN
piliMSH_mshO
piliMSH_mshP
piliMSH_mshQ
piliTap_tapB
piliTap_tapC
piliTap_tapD/pilD
piliTap_tapF
piliTap_tapM
piliTap_tapN
piliTap_tapO
piliTap_tapP
piliTap_tapQ
piliTap_tapT
piliTap_tapU
piliTap_tapV
piliTap_tapW
piliTap_tapY1
piliTap_tapY2
piliTap_tapZ
piliTap_tppA
piliTap_tppB
piliTap_tppC
piliTap_tppE
piliTap_tppF
fimbriae_fimA
fimbriae_fimC
fimbriae_fimD
fimbriae_fimE
fimbriae_fimF
Species
A. caviae
A. veronii
A. veronii AMC34
unique
A. dhakensis
A. hydrophila
A. enteropelogenes
A. jandaei
A. taiwanensis
A. sanarelli

Figure S4. Presence of antimicrobial resistance genes in Aeromonas isolates described in this work. Maximum likelihood phylogenetic tree based on variation in the core genome of isolates (left) with clusters representing species (coloured bar, insert key) and the presence (grey bar) of each gene (top) from ten classes of antimicrobial.

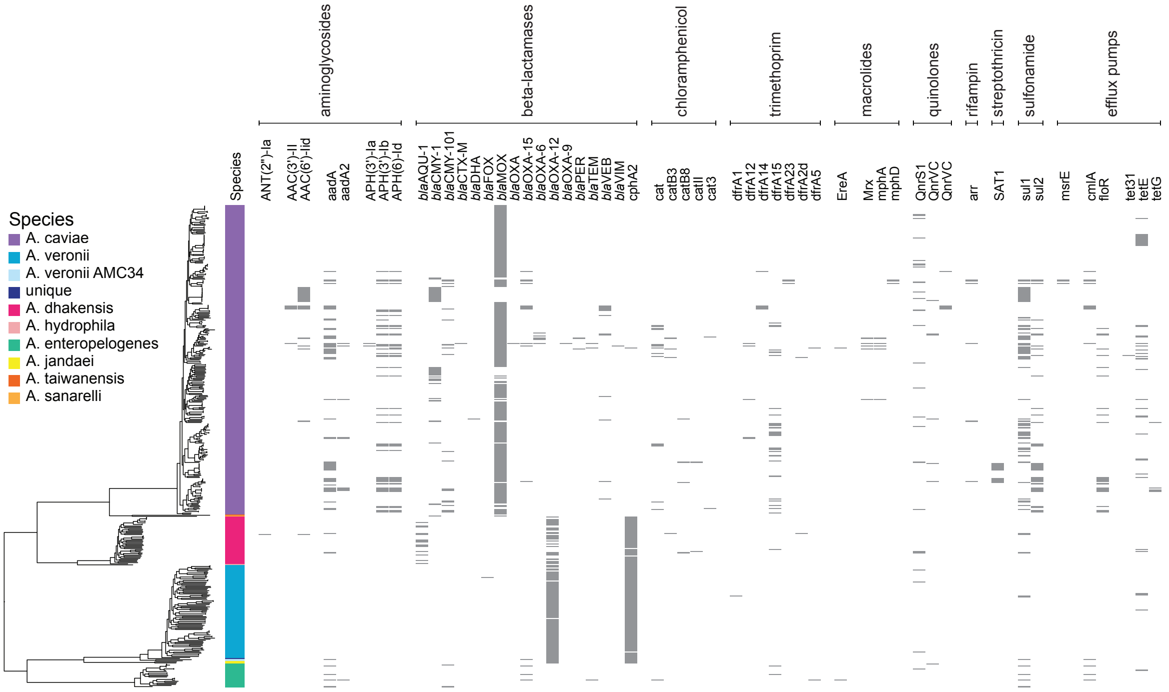

Figure S5. Workflow and exclusion criteria for samples analysed of species and putative virulence gene association with MSD.

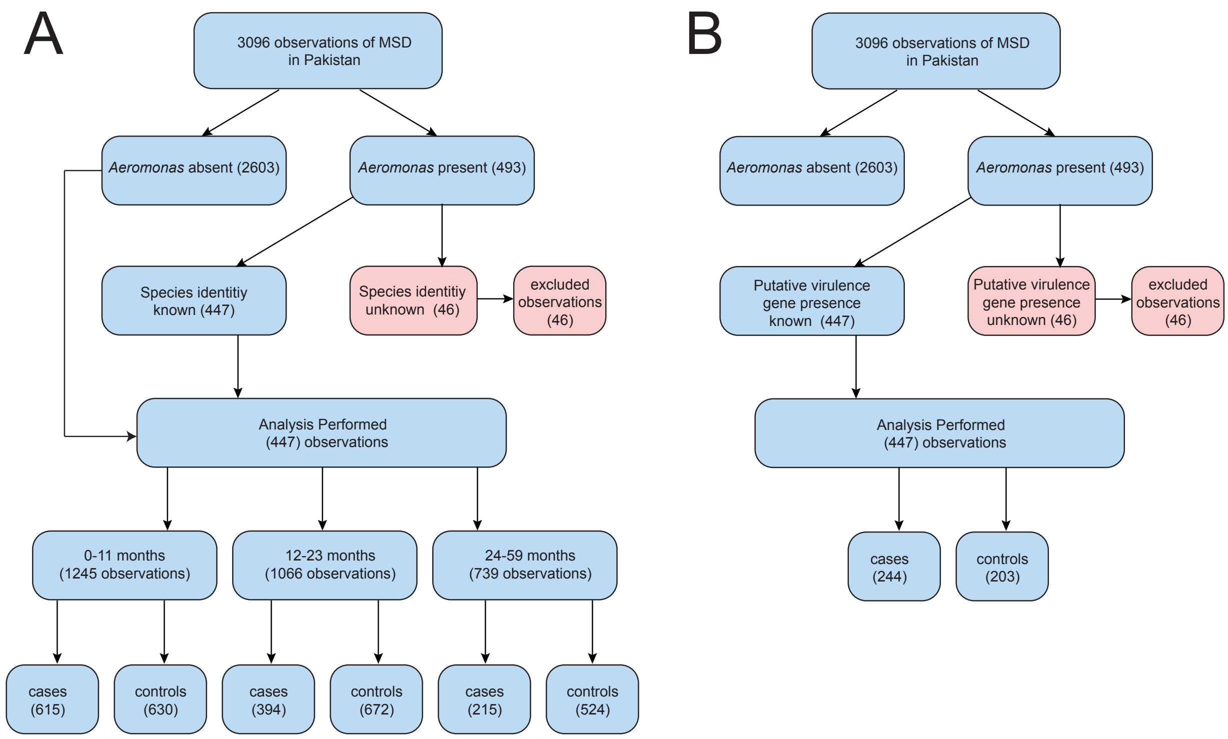

Table S1 **List of strains and sequence data Accession.**
Available for download as an excel file.

Table S2 **Average Nucleotide Identity (ANI) of strains investigated in this study.**
Available for download as an excel file.

Table S3 **Association of each species with moderate to severe diarrhea**

Species	Diarrheal isolates	Total Isolates
A. caviae	153 (53%)	287
A. veronii	46 (53%)	86
A. dhakensis	26 (59%)	44
A. enteropelogenes	12 (55%)	22
A. jandaei	1 (50%)	2
A. veronii AMC34	2 (100%)	2
A. hydrophila	1 (100%)	1
A. taiwanensis	1 (100%)	1
A. sanarellii	1 (100%)	1
unique	1 (100%)	1
	244 (55%)	447

Table S4. Summary table of presence/absence of virulence genes and AMR genes.

Table S5. logistic regression analysis to test for association candidate virulence genes with MSD. Candidate virulence genes that were universally present or had a frequency of less than 5 were excluded from the analysis and a chi square test of association was carried out on the remaining 136 genes.

Table S6. Association of putative virulence genes with MSD. Multivariate analysis was carried out for 34 candidate virulence genes with a p-value of less than or equal to 0.2 in a chi square test of association. Four models were constructed using a backward elimination process; a multivariate model adjusted for the presence of all other genes, a model additionally adjusted for presence of all other pathogens detected in the original study; or additionally adjusted for sociodemographic characteristics, and finally a model adjusted for all other genes, sociodemographic factors, and presence of other pathogens. In this parsimonious model genes that had a p-value >0.05 were removed in a stepwise fashion in order to arrive at the best fitting model with the fewest variables and therefore ORs and p-values that were removed are marked '-'.

File S1. FASTA file of sequences used as representative virulence and AMR genes
